# Supplementary material for: A Multimarker Model for Aberrant Cardiac Geometry after Preeclampsia
Source: J Clin Med. 2022 Mar 29;11(7):1900. doi: 10.3390/jcm11071900 (PMC8999797; doi:10.3390/jcm11071900)
Supplement: Supplementary file 1 [file jcm-11-01900-s001.zip › jcm-1588602-supplementary.pdf]

Supplemental Material

Table S1. Scientific rationale of markers

Markers	Scientific rationale
**SBP (mmHg)**	Women with a history of PE have an almost fourfold increased risk to develop chronic hypertension later in life [1]. Several studies have demonstrated the positive association between SBP and CVD [2]. Hypertension activates an adaptive mechanism of the heart to compensate for elevated pressure which can result in left ventricle hypertrophy [3].
**PV (mL/BSA)**	The increased cardiovascular risk in preeclamptic women may be explained by effect a low plasma volume (LPV) reflecting latent hypertension, [4] The exact mechanism between LPV and hypertension is not defined yet. LPV is reflected as diminished cardiac preload triggering the sympathetic nerves system to increase blood pressure, but with the unfavorable effects on shear stress and cardiac remodeling [5].
**CRP (mg/L)**	In the course towards CVD, inflammatory process are thoug to play in the atherosclerotic process, endothelial damage and microvascular disfunction. Several plasma markers of inflammation have been investigated as potential tools to predict CV risk. [6]. CRP plays an important role in the innate immune response, besides that this marker is a strong predictor of cardiovascular events [7,8].
**Fibrinogen (g/L)**	Fibrinogen is a coagulation factor and an acute-phase inflammatory marker. Fibrinogen deposition in the vessels is the main cause of occlusion and thickening of the walls. This deposition can induce atherosclerosis and stimulate the growth of plaques [9]. Research has shown a positive correlation between an increased plasma fibrinogen levels and CVD [10].
**Uric acid (mmol/L)**	Several studies suggest a high serum uric acid is a risk factor for CVD [11] this could be because uric acid is reacting with nitrogen oxide (NO) in a rapid irreversible reaction resulting in the formation of 6-aminouracil and the depletion of the vasodilating substance NO and in this way leading to vasoconstriction [12].

ATIII, antithrombin; CRP, C-reactive protein; CVD, cardiovascular disease; LPV, low plasma volume; NO, nitrogen oxide; PV, plasma volume; SBP, systolic blood pressure.

1. Bellamy, L.; Casas, J.-P.; Hingorani, A.D.; Williams, D.J. Pre-eclampsia and risk of cardiovascular disease and cancer in later life: systematic review and meta-analysis. *Bmj* **2007**, *335*, 974.
2. Chung, E.; Leinwand, L.A. Pregnancy as a cardiac stress model. *Cardiovasc Res* **2014**, *101*, 561-570, doi:10.1093/cvr/cvu013.
3. Kannel, W.B. Blood pressure as a cardiovascular risk factor: prevention and treatment. *JAMA* **1996**, *275*, 1571-1576.
4. Sagie, A.; Larson, M.G.; Levy, D. The natural history of borderline isolated systolic hypertension. *N Engl J Med* **1993**, *329*, 1912-1917, doi:10.1056/NEJM199312233292602.
5. MacMahon, S.; Peto, R.; Cutler, J.; Collins, R.; Sorlie, P.; Neaton, J.; Abbott, R.; Godwin, J.; Dyer, A.; Stamler, J. Blood pressure, stroke, and coronary heart disease. Part 1, Prolonged differences

in blood pressure: prospective observational studies corrected for the regression dilution bias. *Lancet* 1990, *335*, 765-774, doi:10.1016/0140-6736(90)90878-9.
6. Ridker, P.M.; Hennekens, C.H.; Buring, J.E.; Rifai, N. C-reactive protein and other markers of inflammation in the prediction of cardiovascular disease in women. *N Engl J Med* 2000, *342*, 836-843, doi:10.1056/NEJM200003233421202.
7. Pradhan, A.D.; Manson, J.E.; Rossouw, J.E.; Siscovick, D.S.; Mouton, C.P.; Rifai, N.; Wallace, R.B.; Jackson, R.D.; Pettinger, M.B.; Ridker, P.M. Inflammatory biomarkers, hormone replacement therapy, and incident coronary heart disease: prospective analysis from the Women's Health Initiative observational study. *JAMA* 2002, *288*, 980-987, doi:10.1001/jama.288.8.980.
8. Du Clos, T.W. Pentraxins: structure, function, and role in inflammation. *ISRN Inflamm* 2013, *2013*, 379040, doi:10.1155/2013/379040.
9. Smith, E.B. Fibrinogen, fibrin and fibrin degradation products in relation to atherosclerosis. *Clin Haematol* 1986, *15*, 355-370.
10. Collaboration, F.S. Plasma fibrinogen level and the risk of major cardiovascular diseases and nonvascular mortality: an individual participant meta-analysis. *JAMA* 2005, *294*, 1799-1809, doi:10.1001/jama.294.14.1799.
11. Fang, J.; Alderman, M.H. Serum uric acid and cardiovascular mortality the NHANES I epidemiologic follow-up study, 1971-1992. National Health and Nutrition Examination Survey. *JAMA* 2000, *283*, 2404-2410.
12. Gersch, C.; Palii, S.P.; Kim, K.M.; Angerhofer, A.; Johnson, R.J.; Henderson, G.N. Inactivation of nitric oxide by uric acid. *Nucleosides Nucleotides Nucleic Acids* 2008, *27*, 967-978, doi:10.1080/15257770802257952.
